# Characteristics of Syrian and Lebanese Diabetes and Hypertension Patients in Lebanon

**DOI:** 10.2174/1876526201810010060

**Published:** 2018-12-24

**Authors:** Shannon Doocy, Emily Lyles, Zeina Fahed, Abdalla Mkanna, Kaisa Kontunen, Gilbert Burnham

**Affiliations:** 1 Department of International Health, Johns Hopkins Bloomberg School of Public Health, Baltimore, MD, USA; 2 International Organization for Migration, Beirut, Lebanon

**Keywords:** Hypertension, Diabetes Mellitus, Chronic Disease, Lebanon, Syria, Refugees

## Abstract

**Background:**

Given the protracted nature of the crisis in Syria, the large caseload of Syrian refugee patients with non-communicable diseases, and the high costs of providing non-communicable disease care, implications for Lebanon’s health system are vast.

**Objective:**

To provide a profile of the health status of diabetes and hypertension patients enrolled in a longitudinal cohort study in Lebanon.

**Methods:**

A longitudinal cohort study was implemented from January 2015 through August 2016 to evaluate the effectiveness of treatment guidelines and an mHealth application on the quality of care and health outcomes for patients in primary health care facilities in Lebanon offering low-cost services serving both Syrian refugees and Lebanese host communities. This paper presents baseline characteristics of enrolled patients, providing an overall health status profile.

**Results:**

Among participants, 11.5% of patients with hypertension and 9.7% of patients with diabetes were previously undiagnosed. Quality of care, measured as the proportion of patients with biometrics reported and whose condition is controlled, is less than ideal and varied by geographic location. Controlled blood pressure measurements were observed in 64.2% of patients with hypertension; HbA1C values indicated diabetes control in 43.5% of the patients.

**Conclusion:**

Differences in diagnostic history and disease control between Syrian and Lebanese patients and across geographic regions were observed, which could be applied to inform strategies aimed at improving diagnosis and quality of care for hypertension and diabetes in primary care settings in Lebanon.

## 1 Introduction

The Syrian Civil War, which began in March 2011, has resulted in widespread displacement with more than 6.5 million Syrians internally displaced and an exodus of over five million refugees to countries throughout the region [1]. Lebanon, with just over one million Syrian refugees and an estimated 183 refugees per 1,000 inhabitants, has the largest number of refugees in relation to its host population worldwide [2]. There are no formal Syrian refugee camps in Lebanon. Rather, refugees are integrated with host communities and reside in a variety of settings including rented accommodations, informal settlements (ITS), staying with host families, and other transitional housing in towns and cities.

Like many countries in the region, Lebanese and Syrian populations are in the late stages of epidemiologic transition from communicable, maternal, neonatal, and nutritional conditions to Non-Communicable diseases (NCDs) [3–7]. Type 2 diabetes prevalence is estimated at 8.8% in Syria and 14.9% in Lebanon [8–14]. Previous publications have estimated regional prevalence of hypertension at 29.5%, prevalence in Syria at 24.9%, and in Lebanon at 28.8% [15–18]. Syrian refugees are no exception to the Middle East’s high NCD burden. The United Nations High Commissioner for Refugees (UNHCR) estimates that 14.6% of adult Syrian refugees in Lebanon have a chronic disease and two similar surveys found that 39.8% and 43.4% of Syrian refugee households in Jordan had at least one household member with a chronic health condition (including hypertension, cardiovascular disease, diabetes, chronic respiratory disease, and arthritis) [18, 19]. While estimates of chronic disease prevalence specifically among Syrian refugees in the region are few, the most recently published rates among adult Syrian refugees in Jordan (2015) estimated 9.7% prevalence for hypertension and 5.3% for diabetes. Comparable figures for Lebanon (published in 2016) are similar at 7.4% for hypertension and 3.3% for diabetes [18–21].

With refugees now accounting for one in four people in Lebanon, the increased burden on the country’s fragmented, privatized, and specialist-driven health system is immense and brings financial demands in addition to amplifying infrastructure, capacity, and resource allocation needs [22, 23]. In response to the Syrian crisis, UNHCR established an inter-agency mechanism with the Lebanese government to coordinate the humanitarian response across all sectors. Delivery of health services for Syrian refugees in Lebanon is based on a primary health care strategy. As of August 2015, Syrian refugees registered with UNHCR could utilize primary healthcare services with subsidized fees at approximately 116 of more than 1,200 existing Primary Healthcare Centers (PHCs)/dispensaries across the country, including existing Ministry of Public Health (MoPH) PHCs [24–30].

On top of the Syrian refugee crisis unfolding, a second crisis has emerged concerning the effect of the refugee influx on Lebanese host communities. The majority (86%) of Syrian refugees in Lebanon reside in communities already hosting 66% of the most vulnerable Lebanese populations [31]. Despite priority setting and investment in local and national stability, striking disparities between Syrian refugee and host community populations in Lebanon, specifically related to vulnerability, health status, and access to health care, have been noted in a number of studies [21, 32–35]. Perceptions of inequitable aid distribution and access to basic services in favor of refugees have furthered tensions between refugee and host communities, contributing to increased instability and challenges to international assistance actors and the Lebanese government alike. Despite Lebanon’s relatively small size geographically, there is immense diversity not only among the various populations residing in the country, but also across governorates including a number of previously noted factors associated with geographic differences in communities’ health care utilization and overall health status [36]. Such dissimilarities present added challenges in appropriately meeting the health needs of both refugee and host communities throughout Lebanon.

Given the protracted nature of the crisis, the high cost of providing NCD care, and the large caseload of Syrian refugees with NCDs, implications for Lebanon’s health system are substantial. In light of this, we undertook a study to evaluate the effectiveness of treatment guidelines and an mHealth application on quality of care and health outcomes for patients in primary care settings. In this paper, we present the baseline characteristics of Syrian refugee and Lebanese patients enrolled in the study, which provides a profile of the health status of patients with type 2 diabetes and hypertension at primary health care facilities offering low-cost services in Lebanon.

## 2 Methods

### 2.1 Study Objectives and Aims

This paper presents an overview of characteristics of Syrian refugee and Lebanese patients enrolled in a longitudinal cohort study implemented from January 2015 through August 2016 in primary health care facilities in Lebanon that serve both populations. The main study had two research aims: (1) to adapt and evaluate the use of existing standards and guidelines for treatment, including counselling, of patients with hypertension and type 2 diabetes (or both); and (2) to evaluate the effectiveness of an mHealth application incorporating the aforementioned guidelines that functioned as a decision support tool for providers.

### 2.2 Participants

**Fig. (1):**
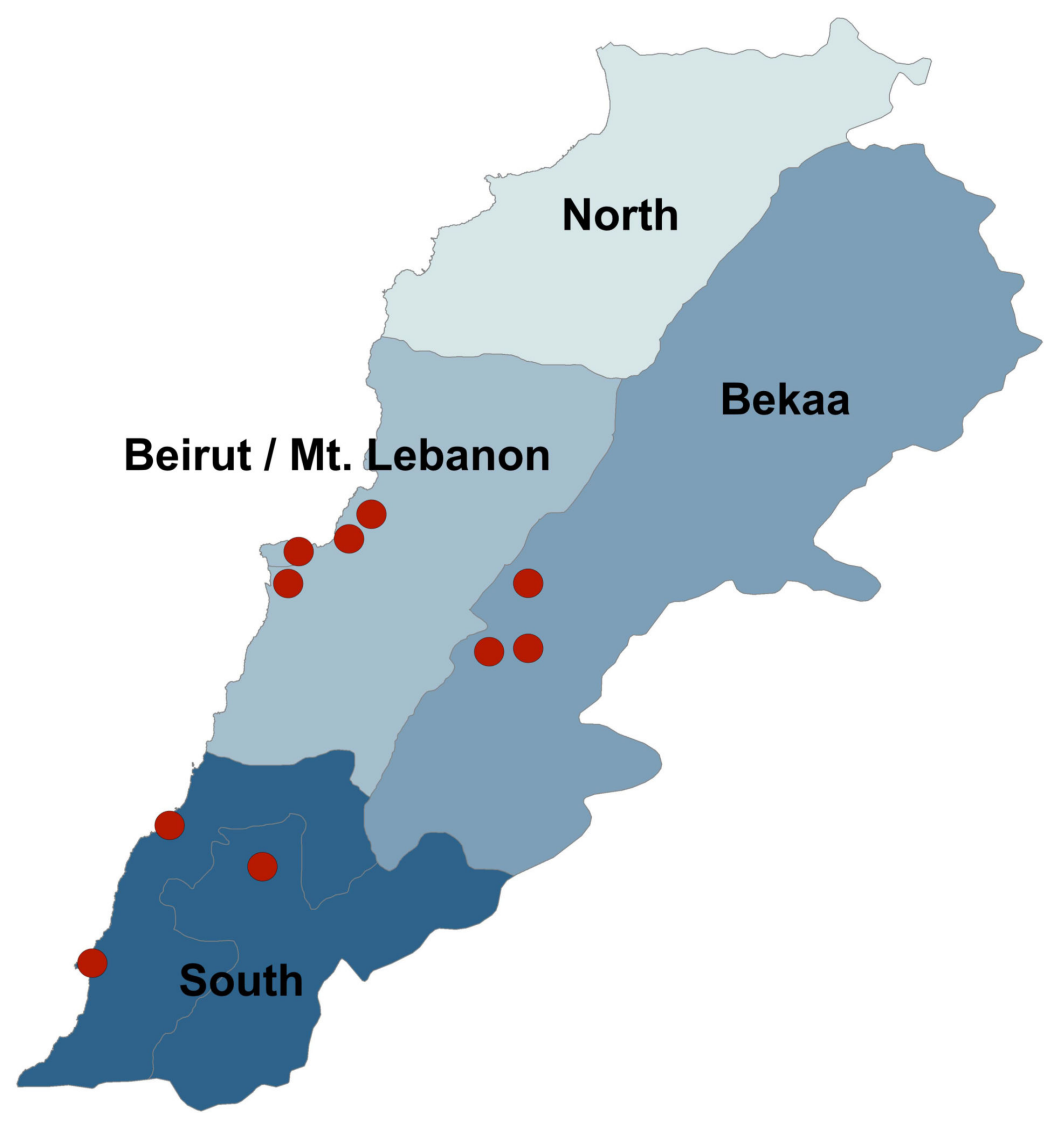
Participating Primary Health Centers by Geographic Area.

The study used a phased introduction of the clinical guidelines and mHealth interventions over a period of 20 months with longitudinal measurement of study outcomes presented elsewhere [37, 38]. Participants consisted of patients at ten health care centers in Lebanon supported by International Organization for Migration (IOM) and International Medical Corps (IMC) in the South, Bekaa, Beirut, and Mount Lebanon governorates Fig. (1). Patients at these locations include Syrian refugees, Lebanese populations, and other nationalities in smaller number, including Iraqi refugees and Armenian populations; individuals of all nationalities were eligible to participate so long as they met inclusion criteria (*i.e.* the sample was not randomly selected). Individuals without hypertension or type 2 diabetes diagnosis, those less than 40 years of age, and adults lacking the capacity to independently participate in interviews were excluded. In this paper, a profile of the health status of enrolled diabetes and hypertension patients at baseline is presented exploring differences in key indicators by geographic location and between Syrian refugee and Lebanese host community patients.

### 2.3 Sample Size

The sample size was estimated for the main study based on reported clinic caseloads, and the anticipated proportions of patients that could be reached by phone (80%) and who would consent to participate (90%). Based on these assumptions, the projected maximum sample size was 1609 participants. Over-estimation of current caseloads resulted in lower actual enrollment; a total of 1010 participants were enrolled and 793 (78%) completed the study. Sample size calculations for the main study were based on the objective of improved quality of care, specifically the proportion of providers that adhere to treatment guidelines, and assumed a baseline rate of 50% for adherence to guidelines (the most conservative rate that would ensure the ability to detect significant differences from all other rates). Sample size calculations were performed using Stata 13, assumed α = 0.05, β = 0.20 (power = 0.80) and were one-sided based on the assumption that quality of care will not decrease as a result of the intervention.

### 2.4 Data Collection

This study was designed using a mixed method approach with qualitative and quantitative data collected throughout. Patients with hypertension or type 2 diabetes were approached at clinics where they received care. If they indicated an interest in participation, a follow-up phone call was made where verbal informed consent was obtained and documented in survey forms. Verbal informed consent was obtained with approval of the Institutional Review Board at The Johns Hopkins Bloomberg School of Public Health because interviews were conducted over the phone, and as such, did not include in-person contact between the study team and patients, and also because of high levels of illiteracy in the Syrian population, especially among older adults and women. Data were collected with tablets using the Magpi mobile data platform by DataDyne LLC (Washington, DC). The baseline interview collected information on demographic characteristics; medical history and recent care seeking behaviors; and knowledge, attitudes, and practices related to diabetes and/or hypertension. Existing surveys from Lebanon and the region were reviewed during the questionnaire design process to identify relevant questions for inclusion that would enable comparison between study findings and national/regional statistics. Medical record reviews were also conducted for each patient following consent and enrollment when information related to provider compliance with guidelines and quality of care; frequency of visits; generic patient outcomes (death and loss to follow-up), and disease-specific patient outcomes (complications and adverse events of hypertension and diabetes) was collected.

### 2.5 Data Analysis

Data were analyzed using Stata 13 (College Station, TX) using descriptive statistics and standard methods for comparison of means and proportions. Blood pressure was monitored among patients with hypertension; the HbA1C test was the preferred measure to monitor patients with diabetes, but when not available, random or fasting blood sugar was used [39–41]. A sequenced process based on patient report, clinical data, and reported prescriptions was applied to assign a uniform diagnosis to patients in cases where reporting was inconsistent over time; eight patients remained with an unclassified diagnosis and were subsequently dropped from final analysis to ensure reliable reporting by condition. Condition control was defined for patients with type 2 diabetes as having HbA1C less than seven percent. For patients with hypertension, condition control was characterized as systolic blood pressure greater than 140 and/or diastolic blood pressure greater than 90mmHg. Differences in patient demographic characteristics as well as diagnostic and care history by population group, geographic region, and condition control status were examined using chi-square and t-test methods. Logistic regression was utilized to determine univariate associations between patient background characteristics and blood pressure control. A multivariate logistic regression model was then developed based on a priori theory adjusting for all covariates of interest. Given the insufficient sample size of patients for whom HbA1C test results were available and the distribution of covariates of interest, regression analysis was not conducted for diabetes control. Financial indicators are presented to the nearest U.S. Dollar (US$) using an exchange rate of 1,507.5 Lebanese Pounds/US$ [42].

## 3 Results

A total of 2,295 individuals were contacted to participate using information provided by study health facilities. Of these, 21.6% (n=498) provided an incorrect phone number, 25.7% (n=592) were unavailable or unable to be reached by phone, 3.9% (n=89) did not meet study criteria, 4.5% (n=104) refused to participate, and 0.1% (n=2) were deceased since the recorded care visit. A total of 1,010 individuals (43.8%) were enrolled and included in the analysis, including 637 Syrian refugees (63.1%), 330 Lebanese (32.6%), and 43 individuals of other nationalities (4.3%).

### 3.1 Sociodemographic Characteristics

More than half (64.0%) of enrolled patients were female and the average age at enrollment was 58.2 (CI: 57.6-58.8) years. Lebanese were significantly older than Syrian refugees (60.5 years vs. 56.8 years, p<0.001) and patients in Beirut/Mt Lebanon were significantly older than in the South and the Bekaa (p<0.001). Lebanese patients had significantly higher educational attainment than refugees with 14.0% completing secondary education or higher as compared to only 6.8% of Syrian refugees (p<0.001); educational attainment also differed significantly by region and was highest in Beirut/Mt Lebanon (p<0.001). Mean household income and expenditures in the month preceding enrolment were US$328 (median US$262, range US$0-2141) and US$672 (median US$619, range US$0-3070), respectively. Income was significantly higher among Lebanese (mean US$386, median US$332) as compared to Syrians (mean US$288, median US$232) and was also significantly higher in Beirut/Mt Lebanon than in the South and the Bekaa. Sale of assets and borrowing, indicative of financial distress, were reported by 13.2% and 57.1% of respondents, respectively with an average debt of US$960 (median US$464, range US$0-10841); borrowing and debt both differed significantly by location (p<0.001). Syrian refugees were 0.31 (CI: 0.19-0.51) times less likely to sell assets and 0.21 times less likely (CI: 0.15-0.27) to borrow money than Lebanese, however, the average debt of Lebanese significantly exceeded that of Syrians (US$1919 vs US$732, p<0.001). Patient sociodemographic characteristics are presented in Table 1.

### 3.2 Diagnostic and Condition History

#### 3.2.1 Hypertension

Among participants, 87.5% (CI: 85.3-89.5) reported having been diagnosed with hypertension at or before the time of enrollment. Hypertension diagnosis was similar by nationality but differed by region with the highest proportion in Beirut/Mount Lebanon and the lowest in the South (94.3% vs 80.0%, p<0.001). Most hypertension participants (88.5%) were diagnosed prior to the facility visit prompting eligibility for study enrollment with an overall average of 7.8 years since initial diagnosis. A significantly higher proportion of Lebanese patients reported a prior diagnosis of hypertension as compared to Syrians (93.2% vs 85.5%, p=0.002) and accordingly had significantly longer average time since diagnosis (9.2 vs 7.2 years, p<0.001). All patients with hypertension reported having ever been prescribed medication for their condition and 98.1% (CI: 96.9-99.0) reported taking medication at the time of interview. Interrupted medication use was relatively low with only 9.3% (CI: 7.3-11.6) of patients prescribed medication for hypertension reporting having stopped the medication for two weeks or more in the three-month period preceding enrollment. Interruption of medication use was significantly higher among Syrian refugees as compared to Lebanese (13.3% vs 2.4%, p<0.001) and was higher in the Bekaa (13.3%) than the South (10.6%) and Beirut/Mount Lebanon (4.3%) (p=0.001). Diagnosis and medication history for hypertension and diabetes is presented in Table 2.

#### 3.2.2 Type 2 Diabetes

Type 2 diabetes was less prevalent than hypertension among study patients with 53.5% (CI: 50.3-56.6) reporting having been diagnosed with type 2 diabetes at or before the time of enrollment. Type 2 diabetes diagnosis was significantly more frequent among Lebanese than Syrians (60.9% vs 49.0%, p<0.001), but similar across geographic regions. Most enrolled patients with diabetes (90.3%) were diagnosed prior to the time of the study enrollment with an overall average of 8.9 years since diagnosis. The mean time since diagnosis was significantly longer among Lebanese as compared to Syrians (10.8 vs 7.8 years, p<0.001). While prior diabetes diagnosis was similar by nationality, significant differences were observed by region; the highest proportion of patients with a prior diagnosis was in the Bekaa (93.9%) and the lowest in the South (85.2%) (p=0.032). Prescription of medication for type 2 diabetes was nearly universal; 99.8% (CI: 98.9-100) of patients with diabetes reported ever being prescribed medication for the condition, of whom 96.4% (CI: 94.4-97.9) reported current medication use at the time of interview. As in patients with hypertension, interrupted medication use for type 2 diabetes was low with only 7.5% (CI: 5.4-10.2) of patients prescribed medication reporting stopping the medication for two weeks or more in the three-month period preceding the enrollment interview. Interrupted medication use was significantly higher among Syrian refugees as compared to Lebanese (10.8% *vs* 2.6%, p<0.001) but was similar across geographic regions.

### 3.3 Disease Control

Control of disease at baseline was determined using measures such as body mass index (BMI), total cholesterol, blood pressure, and HbA1C recorded in patient records (Tables 3 and 4). Reporting varied across the ten study clinics for BMI and cholesterol, resulting in data too incomplete to characterize the study population and sample sizes too small for comparison by region and nationality. Only 7.7% (CI: 6.0-9.7) of patients had a BMI measurement recorded at baseline, among which the average BMI was 33.5 kg/m^2^ (CI: 31.9-35.1; median=32.8). Among those with BMI reported, 7.5% were normal, 22.4% overweight, and 70.1% obese. Fewer than one-third (30.2%, CI: 27.2-33.4) of patients had total cholesterol values recorded. Cholesterol values were recorded most commonly in Beirut/Mt Lebanon (46.2%) and least in the Bekaa (12.2%); Lebanese patients were significantly more likely to have cholesterol values reported compared to Syrians (44.5% vs 29.4%, p=0.003). The average total cholesterol value was 204mg/dL (CI: 198-210; median=202); 46.4% had cholesterol levels in the goal range (<200mg/dL), 33.1% were in the somewhat elevated range (200-239mg/dL) and 20.5% were substantially increased (≥240mg/dL).

#### 3.3.1 Hypertension

**Fig. (2):**
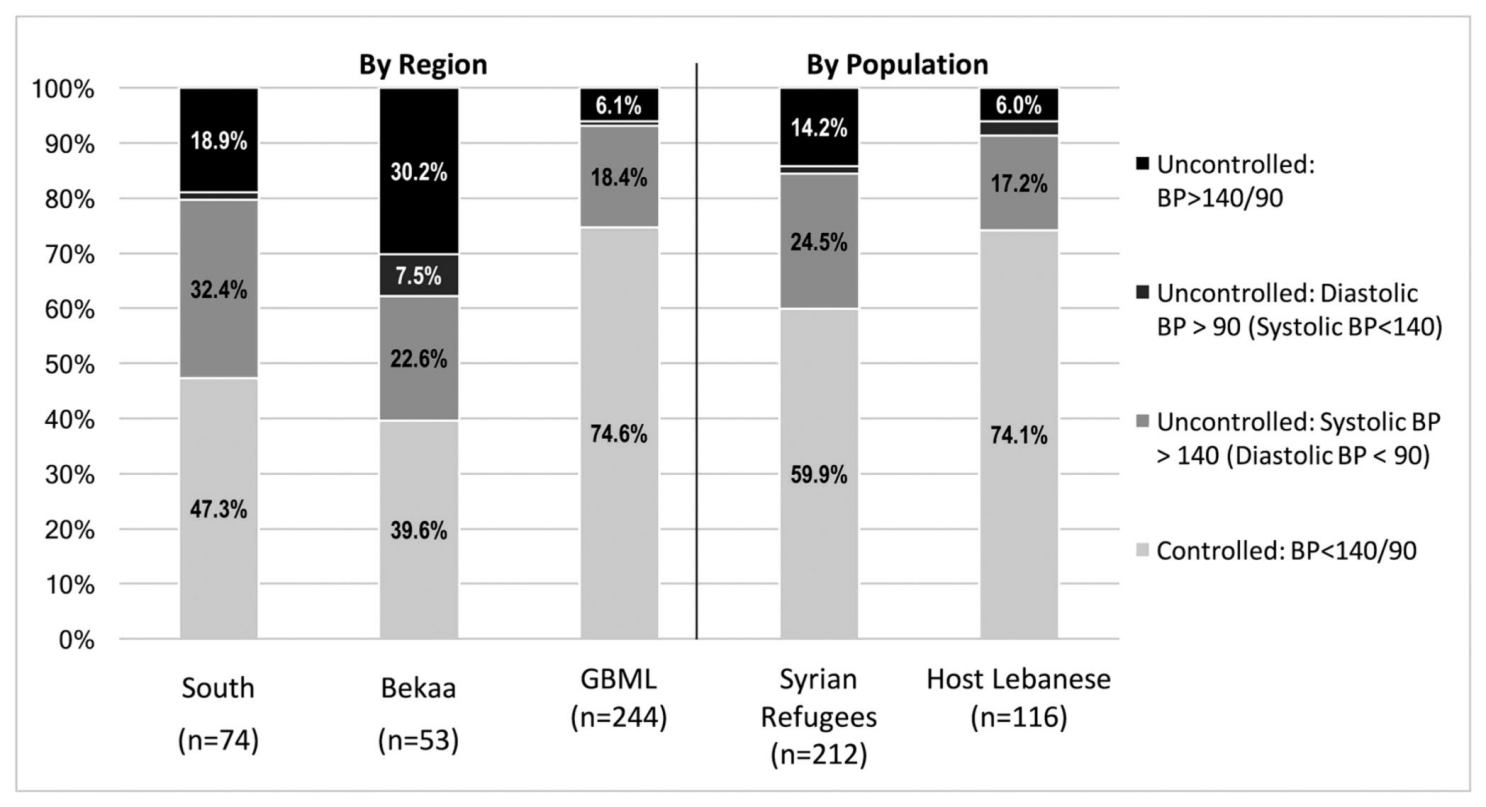
Blood Pressure Control Classification Among Patients with Hypertension.

Blood Pressure (BP) was recorded for 49.1% (CI: 45.5-52.8) of patients with hypertension at baseline, and reporting was significantly higher in Beirut/Mount Lebanon (78.5%) as compared to the South (35.9%) and the Bekaa (22.3%) (p<0.001), but similar by population group (p=0.550). Average systolic BP among patients with hypertension was 138mmHg (CI: 136-140; median=140) and average diastolic blood pressure was 81mm (CI: 79-82; median=80). Overall, 64.2% (CI: 59.0-69.9) of patients with hypertension were classified as having normal BP (lower than 140/90mmHg). Uncontrolled BP was separated into three categories based on both systolic and diastolic readings; 21.8% (CI: 17.7-26.4) of patients with hypertension had systolic BP>140mmHg but diastolic BP <90mmHg (elevated systolic BP); 1.9% (CI: 0.8-3.8) had diastolic BP>90mmHg but systolic BP <140, (elevated diastolic BP), and 12.1% (CI: 9.0-15.9%) had BP above 140/90mmHg (elevated overall BP). Differences in BP classification were statistically significantly different across geographic regions; patients with hypertension in Beirut/Mt Lebanon were most likely to have normal BP (74.6%) at enrollment as compared to patients in the South (47.3%) and the Bekaa (39.6%) (p<0.001). In addition, Lebanese patients with hypertension were significantly more likely to have normal BP than Syrian patients (74.1% vs 59.9%, p=0.028) (Fig. 2).

#### 3.3.2 Diabetes

Among enrolled patients with diabetes, 37.6% (CI: 33.2-42.2) had blood glucose results (including HbA1C, Random Blood Sugar (RBS), and/or Fasting Blood Sugar (FBS)) reported in health facility records at baseline. Similar to blood pressure, blood glucose reporting was significantly higher in Beirut/Mount Lebanon (56.4%) than in the South (21.9%) and the Bekaa (14.8%) (p<0.001), though unlike blood pressure, blood glucose reporting was higher among Lebanese (48.6%) than among Syrian refugee patients (37.3%) (p=0.039). HbA1C was the most commonly used test, for which results were reported for 38.9% (CI: 33.7-44.4) of patients with diabetes; results of HbA1C, RBS and FBS testing are presented in Table 4. Average HbA1C among patients with diabetes was 7.3% (CI: 7.0-7.5; median=7.0). Overall 43.5% (CI: 36.0-51.3%) of patients with diabetes were classified as having controlled diabetes (HbA1C<7%) and 56.5% (CI: 48.7-64.0%) were classified as uncontrolled. No significant difference in control was observed by nationality; however, patients in the Bekaa were significantly less likely to have HbA1C results indicating diabetes control (19.0%) compared to the South (43.5%) and Beirut/Mt Lebanon (55.9%).

### 3.4 Odds of Hypertension Control

Factors associated with control of hypertension were assessed in univariate and multivariate models; however, few significant associations were observed (Table 5). In univariate models for hypertension, age, nationality, region, residence location, and crowding (an economic status proxy) were significantly associated with hypertension control, but only nationality and region remained significant after accounting for other variables in the multivariate model. Syrian refugee patients with hypertension were 2.4 (CI:1.3-4.6) times more likely to have uncontrolled blood pressure than Lebanese and those receiving care in the South were 3.7 (CI: 1.3-10.3) times more likely to have uncontrolled blood pressure than those in Beirut/Mt Lebanon.

## 4 Discussion

The capacity of Lebanon’s health system to support the needs of the Syrian refugee population is becoming increasingly strained despite substantial investment and widespread effort to build the capacity of public institutions [22, 23]. Among patients with hypertension and diabetes in this study, Syrian refugees were significantly less likely to have had a prior diagnosis (*i.e.* to be diagnosed at the visit that led to their study enrollment) for both conditions. This may be tied to the older average age of Lebanese patients enrolled in the study as compared to Syrian refugee patients, but may also reflect poor access or quality of care while in Syria and/or low quality or restricted access to care in Lebanon. Previous findings concerning care-seeking rates among Syrian refugees vary with one study indicating that more than half of households that have members with NCDs are unable to access medicines or related NCD services, while another estimated care-seeking rates at 81% for hypertension and 88% for diabetes [18, 20]. The proportion of individuals with hypertension and diabetes that reported seeking care in the later study was significantly lower among refugees as compared to Lebanese host community members [21]. This disparity is a likely contributor to the higher proportion of new hypertension and diabetes diagnoses observed among Syrian patients as compared to Lebanese in our sample.

In spite of subsidized care available to Syrian refugees for a fee of 3,000 to 5,000 LBP (approximately US$2 to US$3) per visit in over 1200 primary health centers and dispensaries throughout Lebanon, an estimated 39% of Syrian refugees are reportedly not receiving needed medical care because of associated costs [19, 25, 27, 30]. Many Lebanese face similar challenges in accessing affordable health services, where slightly more than half have no formal health insurance coverage [23, 43]. The less affluent and uninsured Lebanese seek care in a network of both public and private health centers, including the MoPH network, which provides reduced cost care for low income Lebanese [25]. Previous findings show that cost is a barrier to care seeking for both Syrian refugees and Lebanese host community members with NCDs; however, a recent survey estimated that 63% of Lebanese with chronic medical conditions sought care at private health centers, demonstrating that Lebanese are more likely to utilize private health facilities when possible, despite the associated higher costs for care [21]. In the same study, both the frequency and amounts of out-of-pocket payments for care were reportedly lower among refugees than host community members, suggesting that current humanitarian support is contributing to maintaining refugee access to NCD care [21].

NCD consultations and medications were offered at low or no cost to both Lebanese and Syrian patients at study health centers and while out-of-pocket payments were not recorded in this study, it is evident from high levels of asset sales and borrowing among refugee patients and debt among refugees and Lebanese alike that financial limitations may affect patients’ care-seeking decisions and ability to afford diagnostics tests and medications, which may not be subsidized. The proportion of newly diagnosed hypertension and diabetes cases was lowest in the Bekaa (for both conditions), as were household incomes and expenditures, while the frequency of borrowing was highest in the Bekaa as compared to Beirut/Mt Lebanon and the South. This is a further suggestion that cost may be a barrier to care, and one that is likely significant enough to delay diagnosis and possibly continuity of care over time. Inability to afford medications may also contribute to the higher rates of interrupted medication use among Syrians that were observed for both hypertension and diabetes patients [32, 44].

The frequency of BMI, cholesterol, blood pressure, and blood sugar reporting was greatest in Beirut/Mt Lebanon, intermediate in the South, and lowest in the Bekaa. When patterns of control were assessed, the proportion of patients with their condition controlled was significantly higher in Beirut/Mt Lebanon as compared to the other regions for cholesterol, hypertension, and diabetes. These findings indicate that access to care and/or quality of care may be higher in Beirut/Mt Lebanon than in the other regions. Improvements in clinical management, such as ensuring that physical and laboratory values are sought and recorded, are a basic practice that should be expanded to improve quality of care. It is possible that cost is a barrier to patients following up on ordered laboratory tests, where such tests are not always subsidized; however, that only 7.7% of participants had BMI reported suggest that inconsistent clinical care practices are also a contributing factor to the low proportion of participants with biometric information recorded in health facility records.

While only three to four clinics participated in the study in each region and results cannot be generalized on a regional basis, participating facilities were perceived as relatively representative of the types of locations where Syrian refugees and less affluent host community Lebanese seek care. When reporting completeness is used as an indicator of quality, our findings suggest that quality of care for NCDs in primary health care settings in Lebanon is low. Considerable efforts are ongoing to bolster the capacity of the Lebanese health system, which will benefit both Syrian refugees and Lebanese. With respect to care for NCDs such as hypertension and diabetes, select health facilities providing subsidized care are mandated to offer services for non-communicable disease prevention, screening, regular follow-up monitoring, and essential medicines; however, little is known about the quality of care for NCDs in MoPH network and other out-of-network health facilities. Further efforts to examine quality of care issues and build capacity to improve quality of care are needed. Disease control could be documented in 64.2% of patients with hypertension and 43.5% of patients with type 2 diabetes [with laboratory results available], further suggesting the need to examine issues such as frequency of care, access barriers, and lifestyle behaviors, all of which contribute to health outcomes in hypertension and type 2 diabetes.

## Limitations

Reliance on self-reported data for many variables of interest may result in bias, particularly for questions about treatment adherence where respondents may be aware of attitudes and perceptions about the importance of adhering to prescribed treatments. Recall of the time of initial diagnosis of hypertension or diabetes may not be accurate. Limiting the study sample to individuals seeking care at study health facilities, while necessary for the main study objectives, is a limitation for the purpose of this research in that it limits representativeness to a small subset of the population of Syrian refugees and host communities in Lebanon who sought care in these locations. The prevalence of hypertension and type 2 diabetes is likely to be much higher in the community than only those recruited for the study without an additional screening of the general population, and persons receiving care from private clinics, pharmacies, or Syrian doctors practicing within the refugee community may well have different findings. As such, results are not necessarily applicable to all patients with hypertension or diabetes who are not receiving care, or who receive care at different types of health facilities or in other geographic areas of Lebanon. This is limiting because outcomes among patients not receiving care could be presumed to be far worse than in patients receiving some level of care; however, results from this research can neither confirm nor preclude those assumptions. Finally, models assessing disease control did not include all factors known to influence the outcome measure such as number and types of medications, co-morbidities and lifestyle behaviors, and would have likely accounted for more variance if additional predictor variables were included.

## Conclusion

Approaches to providing health care to refugees with hypertension and type 2 diabetes are needed that will improve access to care and the quality of care provided. Observed differences between Syrian and Lebanese patients and between patients in different regions could inform strategies aimed at improving diagnosis and quality of care for hypertension and diabetes in primary care settings in Lebanon. Findings from this study suggest that undiagnosed cases of hypertension and type 2 diabetes are not uncommon and that quality of care, as measured by the proportion of patients with recorded biometrics and whose condition is controlled, is less than ideal and varied by geographic location. While efforts to improve quality of care for NCDs, such as the MoPH’s recent update of treatment guidelines, are underway, significant challenges remain to realizing widespread improvements in quality of care for NCDs in primary health facilities. Engaging professional societies to support efforts to ensure consistency and completeness of care for patients with NCDs and additional quality improvement assurance strategies are complementary approaches that could be used to increase uptake of MoPH treatment guidelines.

Improving the treatment of hypertension and diabetes is critical for preventing complications and reducing the need for tertiary care, both of which can be devastating to vulnerable households. Continued efforts to build capacity of health service providers to manage common NCDs such as diabetes and hypertension and ensuring access to affordable consultations, diagnostic testing, and medicines are critical components to addressing the increasing burden of NCDs in Lebanon and improving the health of populations with chronic medical conditions such as diabetes and hypertension.

## Figures and Tables

**Table 1:** Baseline demographic characteristics of enrolled Syrian and Lebanese patients with type 2 diabetes and hypertension.

		Overall (n=1,010)		By Region						*P-value* for regional comparison^a^	By Population				*P-value* for population comparison^a^
				South (n=265)		Bekaa (n=394)		GB/ML (n=351)			Syrian (n=637)		Lebanese (n=330)		
		Point	95% CI	Point	95% CI	Point	95% CI	Point	95% CI		Point	95% CI	Point	95% CI	
**Demographic Characteristics**															
**Sex**															
Male		36.0%	(33.1-39.1%)	34.7%	(29.0-40.8%)	33.5%	(28.9-38.4%)	39.9%	(34.7-45.2%)	0.169	33.0%	(29.3-36.8%)	42.1%	(36.7-47.7%)	0.005
Female		64.0%	(60.9-66.9%)	65.3%	(59.2-71.0%)	66.5%	(61.6-71.1%)	60.1%	(54.8-65.3%)		67.0%	(63.2-70.7%)	57.9%	(52.3-63.3%)	
**Age**	Median	57		56		57		59			56		60		
	Mean	58.19	(57.56-58.82)	56.9	(55.66-58.13)	57.66	(56.67-58.65)	59.75	(58.66-60.85)	0.001	56.8	(56.03-57.56)	60.51	(59.38-61.64)	<0.001
**Highest Level of Education Completed**															
Less than Primary		67.5%	(64.5-70.3%)	71.7%	(65.9-77.0%)	73.5%	(68.8-77.8%)	57.6%	(52.2-62.8%)	<0.001	70.9%	(67.2-74.4%)	62.3%	(56.8-67.6%)	0.002
Primary		23.0%	(20.4-25.7%)	21.9%	(17.1-27.4%)	20.2%	(16.3-24.5%)	27.1%	(22.5-32.0%)		22.3%	(19.1-25.8%)	23.7%	(19.2-28.7%)	
Secondary		7.0%	(5.5-8.8%)	5.3%	(2.9-8.7%)	4.6%	(2.7-7.2%)	11.1%	(8.0-14.9%)		4.9%	(3.3-6.8%)	10.6%	(7.5-14.5%)	
University or higher		2.5%	(1.6-3.6%)	1.1%	(0.2-3.3%)	1.8%	(0.7-3.6%)	4.3%	(2.4-7.0%)		1.9%	(1.0-3.3%)	3.3%	(1.7-5.9%)	
**Marital Status**															
Married		81.3%	(78.7-83.6%)	81.1%	(75.9-85.7%)	84.0%	(80.0-87.5%)	78.4%	(73.7-82.5%)	0.014	81.6%	(78.4-84.6%)	79.4%	(74.6-83.6%)	0.006
Widowed		14.6%	(12.4-16.9%)	14.7%	(10.7-19.6%)	12.7%	(9.6-16.4%)	16.5%	(12.8-20.8%)		15.7%	(13.0-18.8%)	13.6%	(10.1-17.8%)	
Never Married		2.8%	(1.8-4.0%)	1.9%	(0.6-4.3%)	1.5%	(0.6-3.3%)	4.8%	(2.8-7.6%)		1.4%	(0.6-2.7%)	5.2%	(3.0-8.1%)	
Divorced		1.4%	(0.8-2.3%)	2.3%	(0.8-4.9%)	1.8%	(0.7-3.6%)	0.3%	(0.0-1.6%)		1.3%	(0.5-2.5%)	1.8%	(0.7-3.9%)	
**Population Group**															
Syrian Refugee		63.1%	(60.0-66.1%)	68.7%	(62.7-74.2%)	71.6%	(66.8-76.0%)	49.3%	(43.9-54.6%)	<0.001					
Host Lebanese		32.7%	(29.8-35.7%)	30.2%	(24.7-36.1%)	26.7%	(22.3-31.3%)	41.3%	(36.1-46.7%)						
Other		4.3%	(3.1-5.7%)	1.1%	(0.2-3.3%)	1.8%	(0.7-3.6%)	9.4%	(6.6-12.9%)						
**Household Economy^b^**															
**Expenditures (past month)**															
**Total Expenditures**	Median	619		567		529		734			618		624		
	Mean	671.5	(645.6-697.4)	651.7	(606.8-696.7)	589.5	(552.8-626.2)	780.7	(730.2-831.2)	<0.001	659.1	(628.2-690.1)	690.0	(641.3-738.6)	0.275
**Income (past month)**															
**Total Income**	Median	262		332		133		398			232		332		
	Mean	326.8	(304.2-349.5)	369.5	(322.6-416.4)	218.2	(192.9-243.6)	415.9	(371.8-460.0)	<0.001	288.2	(265.9-310.5)	385.8	(336.4-435.3)	<0.001
**Economic Coping Strategies (in past 3 months)**															
**Sold assets to pay for HH expenses**		13.2%	(11.2-15.4%)	13.2%	(9.4-17.9%)	11.7%	(8.7-15.3%)	14.8%	(11.3-19.0%)	0.568	17.1%	(14.3-20.3%)	6.1%	(3.7-9.2%)	<0.001
**Borrowed money to pay for HH expenses**		57.1%	(54.0-60.2%)	58.1%	(51.9-64.1%)	62.8%	(57.9-67.6%)	49.9%	(44.5-55.2%)	<0.001	71.5%	(67.9-75.0%)	34.5%	(29.4-39.9%)	<0.001
**Debt Currently Owed**	Median	464		398		431		531			398		663		
	Mean	960.1	(836.0-1084.1)	897.3	(646.5-1148.0)	796.2	(635.3-957.1)	1235.2	(981.6-1488.8)	0.011	732.0	(631.7-832.2)	1919.1	(1452.6-2385.5)	<0.001

GB/ML = Greater Beirut/Mount Lebanon; 95% CI = 95% confidence interval

a Independent t-test for continuous variables, Chi-square test for categorical variables

b all financial figures presented in US$

**Table 2:** Diagnosis and medication history of enrolled Syrian and Lebanese patients with type 2 diabetes and hypertension

	Overall (n=1,010)		By Region						*P-value* for regional comparison^a^	By Population				*P-value* for population comparison^a^
			South (n=265)		Bekaa (n=394)		GB/ML (n=351)			Syrian (n=637)		Lebanese (n=330)		
	Point	95% CI	Point	95% CI	Point	95% CI	Point	95% CI		Point	95% CI	Point	95% CI	
**Hypertension (HT) History**														
**Ever diagnosed with HT^b^**	87.5%	(85.3-89.5%)	80.0%	(74.7-84.6%)	86.6%	(82.8-89.8%)	94.3%	(91.3-96.5%)	< 0.001	86.7%	(83.8-89.2%)	89.4%	(85.6-92.5%)	0.221
**HT diagnosis at most recent visit^c^**	**n=803**		**n=196**		**n=312**		**n=295**			**n=509**			**n=265**	
At most recent visit	11.5%	(9.3-13.9%)	14.8%	(10.1-20.6%)	8.3%	(5.5-12.0%)	12.5%	(9.0-16.9%)	0.064	14.5%	(11.6-17.9%)	6.8%	(4.1-10.5%)	0.002
No, diagnosed before most recent visit	88.5%	(86.1-90.7%)	85.2%	(79.4-89.9%)	91.7%	(88.0-94.5%)	87.5%	(83.1-91.0%)		85.5%	(82.1-88.4%)	93.2%	(89.5-95.9%)	
**Years since HT diagnosis^d^**	**n=750**		**n=178**		**n=291**		**n=281**			**n=470**			**n=253**	
Median	6		5		6		6			5		7		
Mean	7.81	(7.34-8.28)	6.98	(6.01-7.96)	7.73	(7.01-8.45)	8.42	(7.62-9.23)	0.069	6.96	(6.39-7.53)	9.19	(8.35-10.03)	< 0.001
**Medication**	**n=873**		**n=206**		**n=337**		**n=330**			**n=543**			**n=293**	
Ever prescribed medication	100%	(99.5-100%)	100%	(98.1-100%)	100%	(98.8-100%)	100%	(98.8-100%)	-	100%	(99.3-100%)	100%	(98.6-100%)	-
Currently taking hypertension medication	98.1%	(96.9-99.0%)	98.9%	(96.0-99.9%)	98.0%	(95.6-99.2%)	97.9%	(95.4-99.2%)	0.701	98.1%	(96.4-99.1%)	98.0%	(95.5-99.4%)	0.960
Stopped taking medicines for 2+ weeks in the past 3 months	9.3%	(7.3-11.6%)	10.6%	(6.5-16.1%)	13.3%	(9.6-17.7%)	4.3%	(2.2-7.3%)	0.001	13.3%	(10.4-16.7%)	2.4%	(0.9-5.1%)	< 0.001
**Diabetes (DM) History**														
**Ever diagnosed with DM^b^**	53.5%	(50.3-56.6%)	52.8%	(46.6-59.0%)	53.3%	(48.2-58.3%)	54.1%	(48.8-59.4%)	0.947	49.0%	(45.0-52.9%)	60.9%	(55.4-66.2%)	< 0.001
**DM diagnosis at most recent visit^c^**	**n=506**		**n=135**		**n=196**		**n=175**			**n=287**			**n=194**	
At most recent visit	9.7%	(7.3-12.6%)	14.8%	(9.3-21.9%)	6.1%	(3.2-10.5%)	9.7%	(5.8-15.1%)	0.032	11.9%	(8.3-16.2%)	6.7%	(3.6-11.2%)	0.062
No, diagnosed before most recent visit	90.3%	(87.4-92.7%)	85.2%	(78.1-90.7%)	93.9%	(89.5-96.8%)	90.3%	(84.9-94.2%)		88.2%	(83.8-91.7%)	93.3%	(88.8-96.4%)	
**Years since DM diagnosis^d^**	**n=448**		**n=111**		**n=179**		**n=158**			**n=247**			**n=178**	
Median	7		7		7		8			6		10		
Mean	8.9	(8.2-9.5)	8.3	(7.0-9.7)	8.8	(7.7-9.8)	9.5	(8.4-10.7)	0.386	7.8	(7.0-8.6)	10.8	(9.6-11.9)	< 0.001
**Medication**	**n=537**		**n=139**		**n=208**		**n=190**			**n=309**			**n=201**	
Ever prescribed medication	99.8%	(98.9-100%)	99.3%	(96.0-100%)	100%	(98.1-100%)	100%	(98.1-100%)	0.242	100%	(98.8-100%)	99.5%	(97.3-100%)	0.215
Currently taking diabetes medication	96.4%	(94.4-97.9%)	95.6%	(90.6-98.4%)	98.0%	(94.9-99.4%)	95.4%	(91.2-98.0%)	0.342	96.5%	(93.7-98.3%)	97.4%	(94.1-99.2%)	0.575
Stopped taking medicines for 2+ weeks in the past 3 months	7.5%	(5.4-10.2%)	10.4%	(5.8-16.8%)	8.2%	(4.7-12.9%)	4.6%	(2.0-8.8%)	0.143	10.8%	(7.5-15.0%)	2.6%	(0.8-5.9%)	0.001

GB/ML = Greater Beirut/Mount Lebanon; 95% CI = 95% confidence interval

a Independent t-test for continuous variables, Chi-square test for categorical variables

b Among all enrolled patients (hypertensive and/or diabetic)

c Among patients ever diagnosed with named condition

d Among patients not diagnosed at most recent study clinic visit.

**Table 3:** Biometrics reported in health facility records of enrolled Syrian and Lebanese patients with type 2 diabetes and hypertension.

	Overall^a^		By Region						P-value for regional comparison^b^	By Population				P-value for population comparison^b^
	(n=870)		South (n=260)		Bekaa (n=279)		GB/ML (n=331)			Syrian(n=637)		Lebanese (n=330)		
	Point	95% CI	Point	95% CI	Point	95% CI	Point	95% CI		Point	95% CI	Point	95% CI	
**BMI^c^**	**n=870**		**n=260**		**n=279**		**n=331**			**n=405**		**n=218**	
Total % patients with BMI measured	7.7%	(6.0-9.7%)	4.2%	(2.1-7.4%)	0%	(0.0-1.3%)	16.9%	(13.0-21.4%)	< 0.001	10%	(7.4-13.5%)	11.9%	(7.9-17.0%)	0.488
	**n=67**		**n=11**		**n=0**		**n=56**			**n=41**		**n=26**		
Mean	33.46	(31.85-35.08)	31.71	(27.26-36.15)	-	-	33.81	(32.04-35.58)	0.339	33.12	(31.00-35.24)	34.01	(31.36-36.65)	0.598
BMI < 25 kg/m2 (normal) - “GOAL”	7.5%	(2.5-16.6%)	9.1%	(0.2-41.3%)	-	-	7.1%	(2.0-17.3%)	0.875	7.3%	(1.5-19.9%)	7.7%	(0.9-25.1%)	0.885
BMI > 25kg/m2 (overweight) - “CAUTION””	22.4%	(13.1-34.2%)	27.3%	(6.0-61.0%)	-	-	21.4%	(11.6-34.4%)		24.4%	(12.4-40.3%)	19.2%	(6.6-39.4%)	
BMI > 30kg/m2 (obese) - “HIGH RISK”	70.1%	(57.7-80.7%)	63.6%	(30.8-89.1%)	-	-	71.4%	(57.8-82.7%)		68.3%	(51.9-81.9%)	73.1%	(52.2-88.4%)	
**Blood Lipid - Cholesterol (mg/dL)**	**n=583**		**n=162**		**n=128**		**n=293**			**n=276**		**n=163**		
% total patients with blood lipid test	30.2%	(27.2-33.4%)	29.2%	(23.8-35.2%)	12.2%	(8.6-16.6%)	46.2%	(40.8-51.8%)	< 0.001	29.4%	(25.0-34.1%)	44.5%	(37.8-51.4%)	0.003
	**n=263**		**n=76**		**n=34**		**n=153**			**n=119**		**n=97**		
Mean	204	(198-210)	218	(207-229)	222	(201-243)	194	(186-200)	< 0.001	204	(196-212)	195	(186-205)	0.167
Cholesterol<200 mg/dL “GOAL”	46.4%	(40.2-52.6%)	35.5%	(24.9-47.3%)	38.2%	(22.2-56.4%)	53.6%	(45.4-61.7%)	0.042	42.9%	(33.8-52.3%)	56.7%	(46.3-66.7%)	0.129
Cholesterol=200-239 mg/dL “CAUTION”	33.1%	(27.4-39.1%)	35.5%	(24.9-47.3%)	35.3%	(19.7-53.5%)	31.4%	(24.1-39.4%)		37.0%	(28.3-46.3%)	27.8%	(19.2-37.9%)	
Cholesterol>240 mg/dL “RISK”	20.5%	(15.8-25.9%)	28.9%	(19.1-40.5%)	26.5%	(12.9-44.4%)	15.0%	(9.8-21.7%)		20.2%	(13.4-28.5%)	15.5%	(8.9-24.2%)	
**Hypertension**	**n=755**		**n=206**		**n=238**		**n=311**			**n=353**		**n=185**		
Total % HT patients with BP measured	49.1%	(45.5-52.8%)	35.9%	(29.4-42.9%)	22.3%	(17.1-28.1%)	78.5%	(73.5-82.9%)	< 0.001	60.1%	(54.7-65.2%)	62.7%	(55.3-69.7%)	0.550
	**n=371**		**n=74**		**n=53**		**n=244**			**n=212**		**n=116**		
Controlled: BP<140/90 mmHg	64.2%	(59.0-69.0%)	47.3%	(35.6-59.3%)	39.6%	(26.5-54.0%)	74.6%	(68.6-79.9%)	< 0.001	59.9%	(53.0-66.6%)	74.1%	(65.2-81.8%)	0.028
Uncontrolled: Systolic BP > 140 mmHg (Diastolic BP < 90)	21.8%	(17.7-26.4%)	32.4%	(22.0-44.3%)	22.6%	(12.3-36.2%)	18.4%	(13.8-23.9%)		24.5%	(18.9-30.9%)	17.2%	(10.9-25.4%)	
Uncontrolled: Diastolic BP > 90 mmHg (Systolic BP<140)	1.9%	(0.8-3.8%)	1.4%	(0.0-7.3%)	7.5%	(2.1-18.2%)	0.8%	(0.1-2.9%)		1.4%	(0.3-4.1%)	2.6%	(0.5-7.4%)	
Uncontrolled: BP>140/90 mmHg	12.1%	(9.0-15.9%)	18.9%	(10.7-29.7%)	30.2%	(18.3-44.3%)	6.1%	(3.5-9.9%)		14.2%	(9.8-19.6%)	6.0%	(2.5-12.0%)	
**Systolic blood pressure**	**n=379**		**n=79**		**n=53**		**n=247**			**n=218**		**n=117**		
Mean (in mmHg)	138	(136-140)	148	(143-153)	150	(144-156)	132	(130-134)	< 0.001	140	(137-143)	133	(130-136)	0.003
BP < 140 mmHg	47.5%	(42.4-52.7%)	26.6%	(17.3-37.7%)	28.3%	(16.8-42.3%)	58.3%	(51.9-64.5%)	< 0.001	43.6%	(36.9-50.4%)	53.8%	(44.4-63.1%)	0.011
BP 140-149 mmHg	20.3%	(16.4-24.7%)	19.0%	(11.0-29.4%)	24.5%	(13.8-38.3%)	19.8%	(15.1-25.4%)		19.3%	(14.3-25.1%)	24.8%	(17.3-33.6%)	
BP 150-159 mmHg	12.7%	(9.5-16.4%)	17.7%	(10.0-27.9%)	9.4%	(3.1-20.7%)	11.7%	(8.0-16.4%)		12.8%	(8.7-18.0%)	12.8%	(7.4-20.3%)	
BP 160-169 mmHg	10.0%	(7.2-13.5%)	13.9%	(7.2-23.5%)	13.2%	(5.5-25.3%)	8.1%	(5.0-12.2%)		11.9%	(7.9-17.0%)	5.1%	(1.9-10.8%)	
BP>170 mmHg	9.5%	(6.7-12.9%)	22.8%	(14.1-33.6%)	24.5%	(13.8-38.3%)	2.0%	(0.7-4.7%)		12.4%	(8.3-17.5%)	3.4%	(0.9-8.5%)	
**Diastolic Blood Pressure**	**n=372**		**n=74**		**n=54**		**n=244**			**n=213**		**n=116**		
Mean (in mmHg)	81	(79-82)	85	(83-88)	88	(84-93)	78	(76-79)	< 0.001	82	(80-83)	78	(76-80)	0.023
BP < 90 mmHg	71.2%	(66.3-75.8%)	44.6%	(33.0-56.6%)	46.3%	(32.6-60.4%)	84.8%	(79.7-89.1%)	< 0.001	68.5%	(61.8-74.7%)	80.2%	(71.7-87.0%)	0.074
BP 90-99 mmHg	17.7%	(14.0-22.0%)	36.5%	(25.6-48.5%)	29.6%	(18.0-43.6%)	9.4%	(6.1-13.8%)		18.8%	(13.8-24.7%)	12.9%	(7.4-20.4%)	
BP 100-109 mmHg	8.9%	(6.2-12.2%)	16.2%	(8.7-26.6%)	14.8%	(6.6-27.1%)	5.3%	(2.9-8.9%)		9.9%	(6.2-14.7%)	6.9%	(3.0-13.1%)	
BP > 110 mmHg	2.2%	(0.9-4.2%)	2.7%	(0.3-9.4%)	9.3%	(3.1-20.3%)	0.4%	(0.0-2.3%)		2.8%	(1.0-6.0%)	0.0%	(0.0-3.1%)	

GB/ML = Greater Beirut/Mount Lebanon; 95% CI = 95% confidence interval; BMI = body mass index; HT = Hypertension; BP = blood pressure (in mmHg)

a Includes Syrian refugee and Lebanese host community patients as well as enrolled patients with other nationalities

b Independent t-test for continuous variables, Chi-square test for categorical variables

c In kg/m^2^.

**Table 4:** Diabetes biometrics reported in health facility records of enrolled Syrian and Lebanese patients with type 2 diabetes and hypertension.

	Overall^a^		By Region						P-value for regional comparison^b^	By Population				P-value for population comparison^b^
	(n=870)		South (n=260)		Bekaa (n=279)		GB/ML (n=331)			Syrian(n=637)		Lebanese (n=330)		
	Point	95% CI	Point	95% CI	Point	95% CI	Point	95% CI		Point	95% CI	Point	95% CI	
**Diabetes**	**n=460**		**n=137**		**n=142**		**n=181**			**n=198**		**n=136**		–
% of diabetes patients with blood test results^c^	37.6%	(33.2-42.2%)	21.9%	(15.3-29.8%)	21.8%	(15.3-29.5%)	61.9%	(54.4-69.0%)	< 0.001	37.3%	(30.6-44.4%)	48.6%	(40.0-57.2%)	0.039
	**n=170**		**n=30**		**n=31**		**n=109**		–	**n=73**		**n=66**		–
Controlled	43.5%	(36.0-51.3%)	43.3%	(25.5-62.6%)	12.9%	(3.6-29.8%)	52.3%	(42.5-61.9%)	< 0.001	45.2%	(33.5-57.3%)	53.0%	(40.3-65.4%)	0.357
Uncontrolled	56.5%	(48.7-64.0%)	56.7%	(37.4-74.5%)	87.1%	(70.2-96.4%)	47.7%	(38.1-57.5%)		54.8%	(42.7-66.5%)	47.0%	(34.6-59.7%)	
**HbA1C**	**n=460**		**n=137**		**n=142**		**n=181**		–	**n=198**		**n=136**		–
% of diabetes patients with HbA1C test	33.3%	(29.0-37.8%)	21.9%	(15.3-29.8%)	14.8%	(9.4-21.7%)	56.4%	(48.8-63.7%)	< 0.001	34.3%	(27.8-41.4%)	47.1%	(38.4-55.8%)	0.057
	**n=153**		**n=30**		**n=21**		**n=102**		–	**n=68**		**n=64**		–
Mean	7.3	(7.1-7.5)	7.5	(6.9-8.1)	8.2	(7.6-8.8)	7.0	(6.8-7.2)	< 0.001	7.3	(6.9-7.6)	7.0	(6.7-7.4)	0.284
HbA1C<7.0%	48.4%	(40.2-56.6%)	43.3%	(25.5-62.6%)	19.0%	(5.4-41.9%)	55.9%	(45.7-65.7%)	< 0.001	48.5%	(36.2-61.0%)	54.7%	(41.7-67.2%)	0.709
HbA1C = 7.0-7.9%	21.6%	(15.3-28.9%)	16.7%	(5.6-34.7%)	14.3%	(3.0-36.3%)	24.5%	(16.5-34.0%)		22.1%	(12.9-33.8%)	21.9%	(12.5-34.0%)	
HbA1C > 8.0%	30.1%	(22.9-38.0%)	40.0%	(22.7-59.4%)	66.7%	(43.0-85.4%)	19.6%	(12.4-28.6%)		29.4%	(19.0-41.7%)	23.4%	(13.8-35.7%)	
**Blood Sugar**	–	–	–	–	–	–	–	–	–	–	–	–	–	–
**Random Blood Sugar (Blood Glucose)**	**n=460**		**n=137**		**n=142**		**n=181**		–	**n=198**		**n=136**		–	
% of diabetes patients with random blood sugar test	9.1%	(6.7-12.1%)	0%	(0.0-2.7%)	0%	(0.0-2.6%)	23.2%	(17.3-30.0%)	< 0.001	7.1%	(3.9-11.6%)	19.9%	(13.5-27.6%)	0.001
	**n=42**		**n=0**		**n=0**		**n=42**		–	**n=14**		**n=27**		–	
Mean	158	(145-171)	–	–	–	–	158	(145-171)	–	163	(137-189)	158	(143-173)	0.712
Random Blood Sugar < 120mg/dL	14.3%	(5.4-28.5%)	–	–	–	–	14.3%	(5.4-28.5%)	–	7.1%	(0.2-33.9%)	14.8%	(4.2-33.7%)	0.477
Random Blood Sugar > 120mg/dL	85.7%	(71.5-94.6%)	–	–	–	–	85.7%	(71.5-94.6%)		92.9%	(66.1-99.8%)	85.2%	(66.3-95.8%)	
**Fasting Blood Sugar (Blood Glucose)**	**n=460**		**n=137**		**n=142**		**n=181**		–	**n=198**		**n=136**		–
% of diabetes patients with fasting blood sugar test	24.8%	(20.9-29.0%)	0.7%	(0.0-4.0%)	16.9%	(11.1-24.1%)	49.2%	(41.7-56.7%)	< 0.001	19.7%	(14.4-25.9%)	36.8%	(28.7-45.5%)	0.003
	**n=114**		**n=1**		**n=24**		**n=89**		–	**n=39**		**n=50**		–
Mean	146	(138-154)	90	–	174	(155-194)	139	(131-147)	0.001	145	(132-158)	137	(126-149)	0.385
Fasting Blood Sugar < 100mg/dL	12.3%	(6.9-19.7%)	100%	(2.5-100.0%)	4.2%	(0.1-21.1%)	13.5%	(7.2-22.4%)	0.013	10.3%	(2.9-24.2%)	16.0%	(7.2-29.1%)	0.431
Fasting Blood Sugar > 100mg/dL	87.7%	(80.3-93.1%)	0%	(0.0-97.5%)	95.8%	(78.9-99.9%)	86.5%	(77.6-92.8%)		89.7%	(75.8-97.1%)	84.0%	(70.9-92.8%)	

GB/ML = Greater Beirut/Mount Lebanon; 95% CI = 95% confidence interval

a Includes Syrian refugee and Lebanese host community patients as well as enrolled patients with other nationalities

b Independent t-test for continuous variables, Chi-square test for categorical variables

c Includes HbA1C, fasting blood sugar, random blood sugar, or any combination of those tests.

**Table 5:** Odds of uncontrolled blood pressure among hypertensive Syrian refugees and host communities in Lebanon.

	Controlled BP	Uncontrolled BP^a^	*P-value* ^b^	Odds of Uncontrolled BP^a^	
	(N= 237)	(N= 133)		Crude OR (95% CI)^c^	Adjusted OR (95% CI)^d^
**Individual Condition/Patient Characteristics**					
**Diagnosed comorbidity** (hypertension & type 2 diabetes)	50.20%	47.40%	0.600	1.12 (0.73,1.71)	0.76 (0.44,1.32)
**Time since diagnosis** (in years)					
< 2 years	9.90%	9.80%	0.316	Reference	Reference
2 - 4.9 years	26.20%	19.60%		1.33 (0.55,3.22)	1.06 (0.37,3.03)
5 - 9.9 years	32.20%	38.40%		0.83 (0.36,1.91)	0.65 (0.24,1.74)
10 - 14.9 years	15.80%	21.40%		0.73 (0.3,1.82)	0.8 (0.28,2.32)
15+ years	15.80%	10.70%		1.47 (0.54,3.95)	1.28 (0.39,4.17)
**Age** (in years)					
40 - 49 years	15.60%	19.70%	0.754	Reference	Reference
50 - 59 years	38.00%	35.60%		1.35 (0.73,2.48)	0.88 (0.39,1.97)
60 - 69 years	29.10%	26.50%		1.39 (0.73,2.64)	0.76 (0.33,1.79)
70+ years	17.30%	18.20%		1.2 (0.59,2.44)	0.69 (0.26,1.84)
**Gender**					
Male	40.90%	29.30%	0.026	Reference	Reference
Female	59.10%	70.70%		0.60 (0.38,0.94)	0.74 (0.41,1.35)
**Population Group**					
Host Community	39.60%	23.20%	0.002	Reference	Reference
Syrian Refugee	60.40%	76.80%		2.17 (1.32,3.57)	2.43 (1.29,4.56)
**Highest Education Level Completed**					
Less than Primary	62.90%	65.40%	0.847	Reference	Reference
Primary	25.30%	24.80%		1.06 (0.64,1.75)	1.03 (0.53,1.99)
Secondary	9.30%	6.80%		1.43 (0.63,3.24)	1.21 (0.41,3.58)
University or higher	2.50%	3.00%		0.88 (0.24,3.19)	0.53 (0.1,2.88)
**Household Characteristics**					
**Region of Residence**					
Beirut/Mt. Lebanon	14.80%	28.60%	< 0.001	Reference	Reference
Bekaa	8.40%	24.80%		0.66 (0.32,1.35)	0.96 (0.4,2.34)
South	76.80%	46.60%		3.19 (1.85,5.48)	3.71 (1.34,10.3)
**Residence Area**					
Urban	80.60%	58.30%	< 0.001	Reference	Reference
Rural	19.40%	41.70%		0.34 (0.21,0.54)	1.01 (0.39,2.57)
**Crowding** ^e^	10.60%	21.10%	0.006	0.44 (0.25,0.8)	0.75 (0.35,1.61)
**Socioeconomic Quartile** ^f^					
Bottom	19.40%	21.80%	0.501	Reference	Reference
2nd	24.90%	27.10%		1.03 (0.55,1.93)	0.77 (0.34,1.75)
3rd	25.70%	28.60%		1.01 (0.55,1.87)	0.61 (0.27,1.4)
Top	30.00%	22.60%		1.49 (0.79,2.8)	0.72 (0.31,1.69)

BP = Blood Pressure; OR = Odds Ratio

a Uncontrolled blood pressure (BP) characterized as systolic BP > 140 and/or diastolic BP>90mmHg

b Independent t-test for continuous variables, Chi-square test for categorical variables

c Bold indicates statistically significant (p < 0.10) findings

d Bold indicates statistically significant (p < 0.05) findings

e 5+ people per sleeping room

f Based on monthly expenditures
